# Design of Highly Adhesive and Water-Resistant UV/Heat Dual-Curable Epoxy–Acrylate Composite for Narrow Bezel Display Based on Reactive Organic–Inorganic Hybrid Nanoparticles

**DOI:** 10.3390/polym12102178

**Published:** 2020-09-24

**Authors:** Jun Hyup Lee

**Affiliations:** Department of Chemical Engineering, Soongsil University, Seoul 06978, Korea; junhyuplee@ssu.ac.kr; Tel.: +82-2-829-8329

**Keywords:** adhesion strength, epoxy–acrylate composite, narrow bezel display, reactive hybrid nanoparticle, water permeability

## Abstract

To attain the narrow bezel characteristic of information displays, functional sealing composite materials should possess high adhesion strength and water barrier performance due to their narrow line widths. In this study, highly adhesive UV/heat dual-curable epoxy–acrylate composites with outstanding water-resistant performance have been proposed using photoreactive organic–inorganic hybrid nanoparticles that can react with an acrylate resin, creating a crosslinked nanoparticle network within the sealing composite. The hybrid nanoparticles consisted of reactive methacrylate groups as a shell and an inorganic core of silica or aluminum oxide, and were facilely synthesized through sol–gel reaction and chemisorption process. The curing characteristics, adhesive strength, and moisture permeability of the proposed sealing composite have been compared to those of a conventional epoxy–acrylate composite containing inorganic silica particles. The composites including hybrid nanoparticles exhibited high UV and heat curing ratios owing to the numerous methacrylate groups on the nanoparticle surface and high compatibility with organic resins. Moreover, the proposed sealing composite showed high adhesion strength and extremely low water permeability due to the creation of densely photocrosslinked network with matrix resins. In addition, the sealing composite exhibited excellent narrow dispensing width as well as relatively low viscosity, suggesting the potential application in narrow bezel display.

## 1. Introduction

Recently, super narrow bezel displays have attracted increasing interest due to the simple fabrication of large-area tiled display and realization of a truly immersive experience. Among various display modes, liquid crystal display has been widely utilized in narrow bezel applications such as notebooks, monitors, televisions, and digital signage. Since the sealing composite materials play a key role in fabricating the liquid crystal device [1,2,3,4,5], the performance of the sealing composite should be improved according to the narrow bezel width of the display. Traditionally, the sealing material is dispensed onto the outer area of substrates, and then cured by heat or ultraviolet (UV) light, thereby offering firm adhesion between top and bottom substrates as well as moisture barrier properties.

In general, the sealing composite materials for information displays include a UV-curable acrylate resin or heat-curable epoxy resin, a photoinitiator, a heat curing agent, and inorganic fillers. Among these constituents, the reactive resin as the main component of the sealing composite has a large influence on the physical and chemical properties of the composite. Especially, heat-curable epoxy resins have good chemical stability, high adhesion strength, and excellent thermal stability, but they need high heat-curing temperature and long processing time [6,7]. In contrast, acrylate resins with photoreactive vinyl end groups show relatively low adhesion and high gas permeation, however they are easy to be cured by UV light during short irradiation time [8,9]. Therefore, this UV-curing system has been widely adopted in industrial applications such as coatings, photoresists, and pressure-sensitive adhesives due to fast curing speed, low processing cost, high chemical stability, and solvent-free procedure at room temperature [10,11,12,13].

The inorganic filler, which is a large part of the sealing composite, not only reduces the material cost of the composite, but also improves the physical properties such as the thermal shrinkage, moisture permeability, and mechanical properties [14,15,16]. In particular, silica and aluminum oxide particles are mostly utilized in various polymer composite materials [17,18,19,20,21,22,23,24]. Since the surface characteristic of the filler particle greatly affects the mechanical properties, the tensile strength can be decreased as a result of an increase in the particle size [17]. Moreover, an increase in filler content and specific area can improve the mechanical properties of the composite. However, an excessive increase in the inorganic filler content can induce severe aggregation of the particles, leading to high viscosity of the composite and inhomogeneous dispersion of filler particles. To prevent aggregation of the inorganic filler particles, their surface can be modified with organic functional groups [8,18,19,20]. Through these modifications, the tensile strength and elongation of the polymer composite were improved. Particularly, the organic modification of aluminum oxide surface increased the initial reaction velocity of the epoxy–amine reaction due to the decrease in glass transition temperature of the epoxy resin [21,22]. Furthermore, the nanoscale inorganic fillers showed a tendency to enhance tensile strength and toughness [23,24].

**Figure 1:**
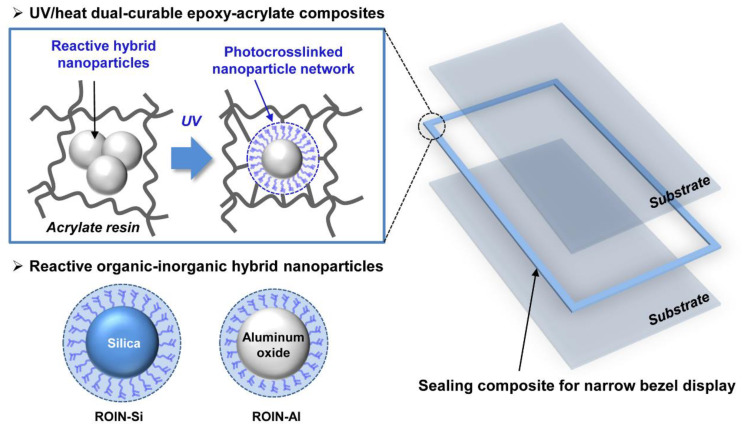
Schematic illustration of UV/heat dual-curable epoxy–acrylate composites embedded with photoreactive organic–inorganic hybrid nanoparticles for narrow bezel display.

In this study, highly adhesive and water-resistant UV/heat dual-curable epoxy–acrylate composites with high curing conversion and narrow dispensing width for potential sealing material in narrow bezel display have been newly fabricated using photoreactive organic–inorganic hybrid nanoparticles. As shown in Figure 1, reactive hybrid nanoparticles consisting of silica or aluminum oxide as core and photoreactive methacrylate groups as shell were synthesized through simple sol–gel reaction and chemisorption. The UV/heat dual-curable epoxy–acrylate composites incorporated with hybrid nanoparticles were prepared with bisphenol A epoxy resin and bisphenol A glycerolate dimethacrylate resin through revolution–rotation mixing process. The embedded photoreactive organic–inorganic hybrid nanoparticles can facilitate the UV curing reaction with acrylate resin due to the presence of numerous methacrylate groups in the surface area of nanoparticles, and thereby improve the compatibility between hybrid nanoparticle fillers and reactive organic resins [25]. The adhesive strength and water barrier performance of the proposed sealing composites were improved by employment of reactive hybrid nanoparticles due to the formation of densely crosslinked nanoparticle network with acrylate resins compared to those of conventional sealing composite including inorganic silica particles. In addition, narrow dispensing width and relatively low viscosity were obtained for new epoxy–acrylate composites owing to high compatibility of nanosized hybrid fillers with reactive resins. It is expected that the new type of epoxy–acrylate composites with photoreactive hybrid nanoscale fillers can be useful as the functional sealing materials for narrow bezel displays due to their advantages of simple preparation, high curing conversions, excellent adhesive strength, extremely low moisture permeability, low viscosity, and narrow dispensing width.

## 2. Materials and Methods

### 2.1. Materials

Tetraethyl orthosilicate (TEOS), 3-(trimethoxysilyl)propyl methacrylate (3-MPMA), methacrylic acid, and adipic acid dihydrazide (ADH) were purchased from Tokyo Chemical Industry Co., LTD (Tokyo, Japan). Fumed silica with an average diameter of 250 nm and bisphenol A glycerolate dimethacrylate (BisGMA) resin were obtained from Sigma-Aldrich (St. Louis, MO, USA). The aluminum oxide nanopowder dispersion with an average particle size of 10 nm (Al_2_O_3_ gamma, 20 wt.%) was purchased from US Research Nanomaterials, Inc. (Houston, TX, USA). The ammonia solution (28–30 wt.%) and sodium hydroxide pellets (NaOH) were received from Samchun Pure Chemical Co., Ltd. (Pyeongtaek, Korea). The bisphenol A type epoxy resin (YD-128) was obtained from Kukdo Chemical (Seoul, Korea). Irgacure 651 was received from Ciba Specialty Chemicals (Basel, Switzerland). Drierite (≥98% CaSO_4_) was obtained from W.A. Hammond Drierite Co., LTD (Xenia, OH, USA). Deionized (DI) water was used for all experiments.

### 2.2. Synthesis of Reactive Organic–Inorganic Hybrid Silica Nanoparticles

The synthesis of reactive hybrid silica nanoparticles (ROIN-Si) was based on the sol–gel method described by Luo et al. [19]. First, ethanol (200 mL), ammonia solution (6 mL), and DI water (16 mL) were mixed in a 500 mL beaker, and the solution was vigorously stirred for 30 min at 25 °C. Then, 12 mL of TEOS was slowly dropped into the beaker, and the solution was stirred for 30 min at 25 °C. Subsequently, 14.5 mL of 3-MPMA was slowly dropped into the beaker, and then the solution was stirred for 10 h at 25 °C and finally, the suspension was aged for 5 h. The product was precipitated several times by repeated centrifugation with steps of 9000 rpm for 20 min. The precipitates were washed several times using DI water and ethanol. The final products were dried in vacuum at 60 °C for 24 h.

### 2.3. Synthesis of Reactive Organic–Inorganic Hybrid Aluminum Oxide Nanoparticles

The reactive hybrid aluminum oxide nanoparticles (ROIN-Al) were synthesized through chemisorption process [26,27]. Methacrylic acid (1120 μL) was dissolved in 500 mL of NaOH solution (1 mM). The solution was vigorously stirred for 30 min at 80 °C. Then, 12.5 g of aluminum oxide dispersion (20 wt.% in water) was poured into the solution, and it was stirred for 30 min at 85 °C. The resulting solution was sonicated in an ultrasonic bath for 60 min at 80 °C. After sonication, the product was cooled at 25 °C and the NaOH was added to increase the pH to 10. The product was precipitated several times by repeated centrifugation with steps of 10,000 rpm for 30 min. The precipitated product was washed with DI water until pH reached 7, and then it was washed several times with acetone. The final product was dried in vacuum at 40 °C for 24 h.

### 2.4. Preparation of UV/Heat Dual-Curable Epoxy–Acrylate Composites Embedded with Reactive Nanoparticles

The UV/heat dual-curable epoxy–acrylate composites were prepared through a revolution–rotation mixing process (AR-100, Thinky, Tokyo, Japan). Table 1 shows the composition of the prepared epoxy–acrylate composites. The acrylate resin of BisGMA and epoxy resin of YD-128 were poured in a mixing container, and then mixed at 2200/800 rpm (revolution/rotation) for 15 min. Subsequently, the fillers were added to the container and then mixed for 15 min. Then, the hardener and photoinitiator were added and mixed for 15 min. Finally, the composite mixture was defoamed for 3 min at 2000 rpm.

### 2.5. Characterization

Fourier-transform infrared (FTIR) spectra were recorded on a spectrometer (FT/IR-460, Jasco, Tokyo, Japan) using KBr disks and the attenuated total reflectance (ATR) mode. The epoxy–acrylate composites were cured at 3 J cm^−2^ of UV energy, and then thermally cured at 120 °C for 1 h on a hot plate. The UV curing of the sealing composites was performed on a UV irradiator (KJPHT-101, KJUV, Incheon, Korea). The morphology and size of ROIN-Si and ROIN-Al nanoparticles were analyzed using field emission scanning electron microscope (FE-SEM, SU-70, Hitachi, Tokyo, Japan) and Zetasizer equipped with dynamic light scattering (Zetasizer Nano ZS, Malvern Instruments, Malvern, UK). The adhesive strength was measured by using a universal testing machine (UTM, Lloyds LR-5K, Lloyd Instruments Ltd., Fareham, UK). The test specimens were pulled off at a speed of 1.3 mm min^−1^. The viscosities of the composites were measured using a viscometer (DV-II + VISCOMETER, Brookfield Ametek, Middleborough, MA, USA). Spindle 64 was used at a shear rate of 10 rpm for 1 min, and the homogeneously mixed composite samples using the aforementioned revolution–rotation mixing process were utilized to measure the viscosity. The narrow dispensing experiment was performed on Janome seal dispenser with dispensing pressure and drawing speed of 60 psi and 1 mm s^−1^, respectively. The microscopic images were obtained by using an optical microscope (OM, BX51, Olympus, Tokyo, Japan). In order to measure the moisture permeability of the sealing composites, the top of a flat vial containing drierite was applied with sealing composites, and attached with cover glass. Then, UV curing with 3 J cm^−2^ and heat curing at 120 °C for 1 h were performed. After the sealed vials were placed in DI water at 120 °C for 1 h, the increased weight of the drierite was measured.

## 3. Results and Discussion

### 3.1. Preparation of Reactive Organic–Inorganic Hybrid Nanoparticles

**Figure 2:**
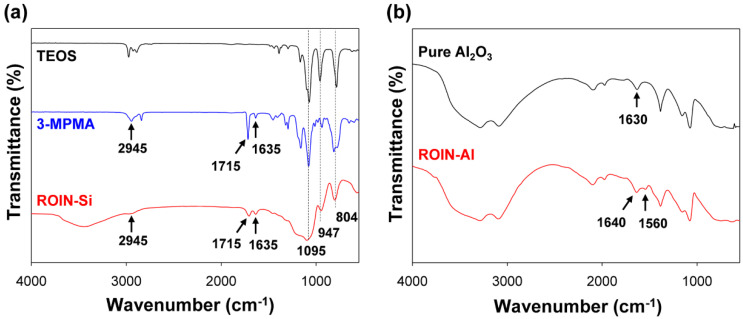
(**a**) FTIR spectra of tetraethyl orthosilicate (TEOS), 3-(trimethoxysilyl)propyl methacrylate (3-MPMA), and reactive hybrid silica nanoparticle. (**b**) FTIR spectra of pure aluminum oxide and reactive hybrid ROIN-Al nanoparticle.

The reactive organic–inorganic hybrid silica nanoparticle was synthesized using the sol–gel method [19]. Figure 2a shows the FTIR spectra of TEOS, 3-MPMA, and ROIN-Si nanoparticle measured using a KBR pellet and ATR mode. The ROIN-Si nanoparticle showed characteristic stretching vibrations of carbonyl (C=O) group, carbon–carbon double bond (C=C), and methylene moiety (CH_2_) at 1715, 1635, and 2945 cm^−1^, respectively [19]. These carbonyl and C=C double bond peaks, which were not observed for TEOS due to the absence of methacrylate functional groups, were ascribed to the surface modification of pure silica particle using 3-MPMA with a photoreactive methacrylate group. In addition, the absorption peaks at 804, 947, and 1095 cm^−1^ were attributed to silica core particle [19]. Thus, it is confirmed that a reactive hybrid silica nanoparticle was successfully synthesized through sol–gel process.

Photoreactive hybrid aluminum oxide nanoparticle was synthesized via a simple chemisorption process [27]. Figure 2b shows the FTIR spectra of pristine aluminum oxide and the reactive hybrid ROIN-Al nanoparticle. The pure aluminum oxide particle showed a low-intensity peak at 1630 cm^−1^, which was ascribed to the dawsonite-like structure created by adsorption of carbon dioxide and water molecules [28]. As a result of surface modification with methacrylic acid, the C=C double bond peak and the asymmetric stretching vibration of the carboxylate (COO–) group corresponding to the methacrylate group were observed at 1640 and 1560 cm^−1^, respectively, suggesting the successful surface modification of pristine aluminum oxide [29,30,31].

**Figure 3:**
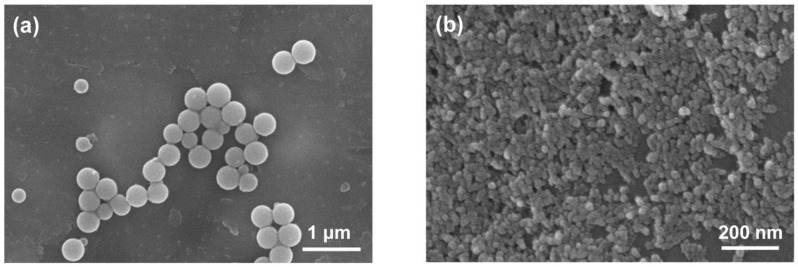
FE-SEM images of (**a**) ROIN-Si and (**b**) ROIN-Al nanoparticles.

**Figure 4:**
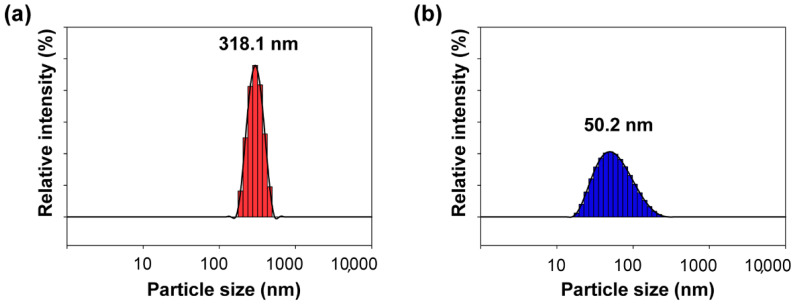
Particle size distributions of (**a**) ROIN-Si and (**b**) ROIN-Al nanoparticles.

Figure 3 shows FE-SEM images of the reactive hybrid ROIN-Si and ROIN-Al nanoparticles. The synthesized surface-modified hybrid nanoparticles had uniform spherical shape with a nanoscale size. The ROIN-Si nanoparticles exhibited larger particle size than ROIN-Al. The detailed particle sizes of ROIN-Si and ROIN-Al nanoparticles are shown in Figure 4. The reactive silica nanoparticles exhibited a relatively large particle size of 318.1 nm, whereas reactive aluminum oxide nanoparticles showed a small particle diameter of 50.2 nm. Since the small nanoparticle size is advantageous for controlling the particle aggregation in the composite, the efficient curing reaction between reactive nanoparticles and acrylate resins can be achieved by a curing process, which can improve the adhesive strength and water barrier performance of the sealing composite. In addition, the average particle sizes of the prepared ROIN-Si and ROIN-Al nanoparticles were larger than those of pristine silica and aluminum oxide nanoparticles with average diameters of 250 and 10 nm, respectively, indicating the formation of an organic shell on an inorganic nanoparticle core.

### 3.2. Viscosity and Narrow Dispensing Characteristics of UV/Heat Dual-Curable Epoxy–Acrylate Composites

**Figure 5:**
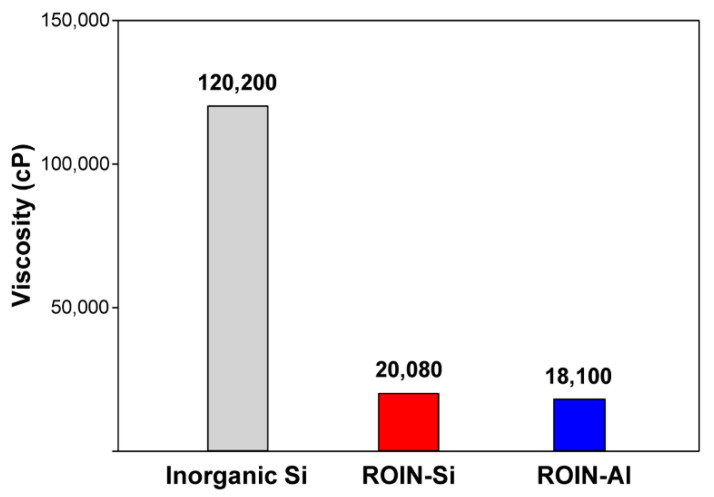
Viscosities of conventional sealing composite with inorganic Si and new epoxy–acrylate composites with ROIN-Si and ROIN-Al nanoparticles.

The viscosity and narrow dispensing width of new UV/heat dual-curable epoxy–acrylate composites containing reactive hybrid nanoparticles were examined. Figure 5 shows the viscosities of conventional and new epoxy–acrylate composites. The conventional sealing composite including inorganic fumed silica particle exhibited the highest viscosity of 120,200 cP. The severe aggregation of Si particles led to high viscosity of composite due to the occluding of the organic polymers in the interparticle voids [32]. In contrast, new epoxy–acrylate composites containing surface-modified silica or aluminum oxide nanoparticles showed a relatively low viscosity of 20,080 and 18,100 cP, respectively. The reactive organic shell of the hybrid nanoparticles can reduce the particle aggregation and improve the compatibility with organic resins, thereby lowering the viscosity of the composite materials [33]. The composite with reactive ROIN-Al nanoparticles showed a relatively lower viscosity than that with ROIN-Si nanoparticles due to the small particle size. Low viscosity of the composite can facilitate efficient dispersion of nanoparticles during mixing process and therefore enhance the mechanical and barrier properties of the composite.

**Figure 6:**
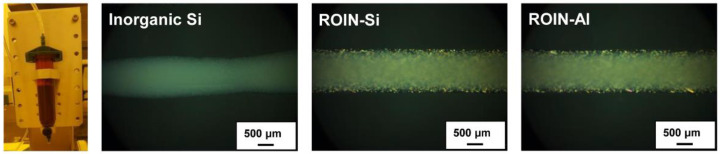
Microscopic images of dispensing lines after drawing the sealing composites with inorganic Si, ROIN-Si, and ROIN-Al nanoparticles between two glass substrates using commercial seal dispenser.

Figure 6 shows the microscopic images of narrow dispensing lines after drawing the sealing composites including reactive silica or aluminum oxide nanoparticles between glass substrates using common seal dispenser. While a non-uniform drawing line was observed for a conventional sealing composite with inorganic silica particles, the epoxy–acrylate composites embedded with reactive hybrid nanoparticles exhibited narrow and uniform dispensing lines with an average line width of about 1.4 mm. The surface modification of inorganic nanoparticles with organic methacrylate groups induces high compatibility with organic resins and low viscosity of the composite, and consequently facile manipulation of narrow dispensing line can be accomplished by using new epoxy–acrylate composites. These results confirm that the proposed sealing composites can afford narrow dispensing performance for narrow bezel display applications.

### 3.3. Curing Behaviors of UV/Heat Dual-Curable Epoxy–Acrylate Composites

**Figure 7:**
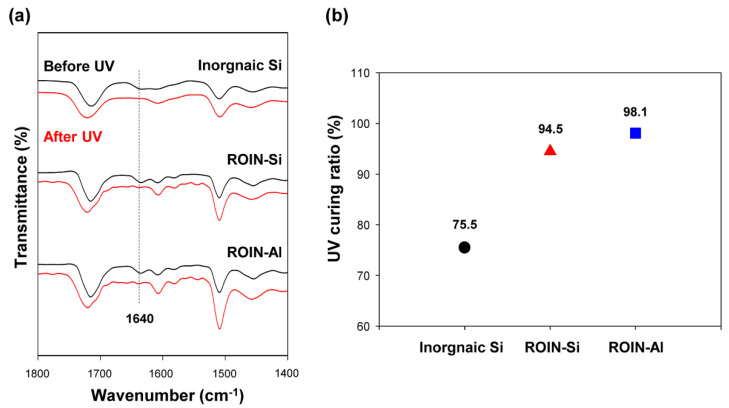
(**a**) FTIR spectra of the epoxy–acrylate composites containing inorganic silica or reactive hybrid nanoparticles before (black) and after (red) UV curing. (**b**) UV curing ratios of the composites with inorganic silica or reactive hybrid nanoparticles.

Figure 7a shows the FTIR spectra of UV/heat dual-curable epoxy–acrylate composites containing conventional inorganic silica or reactive hybrid nanoparticles before and after UV curing. In all epoxy–acrylate composites, the stretching vibration of carbon–carbon double bond at around 1640 cm^−1^ was dramatically reduced after UV irradiation irrespective of filler type. In order to examine the detailed difference in the UV curing conversions of the composites, the UV curing ratio was calculated by Equation (1) based on the peak area of methacrylate group at 1640 cm^−1^. The peak area decreased after UV treatment due to the photocrosslinking reaction of vinyl groups.

(1) $$\mathrm{Curing}\;\mathrm{ratio}\;\left(\%\right)=\frac{peak\;area\;before\;curing-peak\;area\;after\;curing}{peak\;area\;before\;curing}\times 100$$

The calculated UV curing ratios for common inorganic silica and reactive hybrid nanoparticles are shown in Figure 7b. While the UV curing ratio of conventional composite with unreactive inorganic silica was a low value of 75.5%, the proposed composites with photoreactive hybrid silica or aluminum oxide nanoparticles showed remarkably high UV curing ratios of 94.5% and 98.1%, respectively. The inorganic silica particles with only hydroxyl terminal groups are not able to react with matrix resins, whereas a large number of methacrylate functional groups in the shell of nanoparticles can promote the multi-curing reactions with acrylate matrix resin, leading to the increase in the UV curing ratio of the composite [34]. Notably, the proposed composite with reactive ROIN-Al nanoparticles showed a relatively high UV curing ratio due to the small particle size and low viscosity compared to that of composite with ROIN-Si nanoparticles.

**Figure 8:**
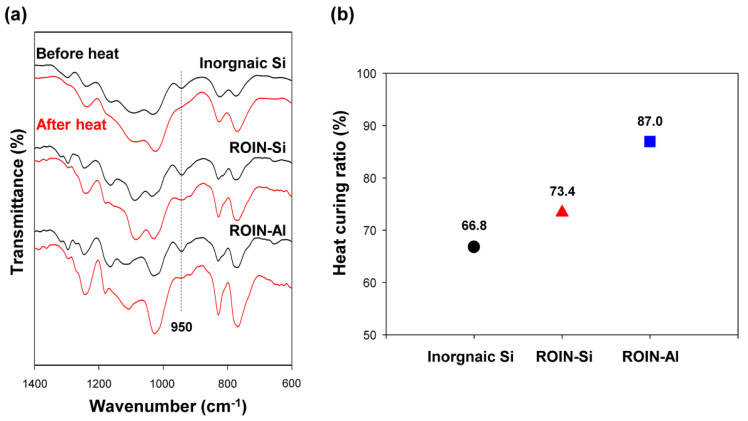
(**a**) FTIR spectra of the epoxy–acrylate composites containing inorganic silica or reactive hybrid nanoparticles before (black) and after (red) heat curing. (**b**) Heat curing ratios of the composites with inorganic silica or reactive hybrid nanoparticles.

Figure 8 shows the FTIR spectra of the epoxy–acrylate composites including inorganic silica or reactive hybrid nanoparticles before and after heat curing. The stretching vibration of the epoxy group at 950 cm^−1^ was greatly reduced after heat curing for all sealing composites, as shown in Figure 8a. The epoxide rings of epoxy resin in the composites were thermally reacted with hardeners bearing amine groups, and thereby the peak area of epoxy group decreased after heat treatment. The heat curing ratios of the composites were calculated based on Equation (1) and displayed in Figure 8b. The surface-modified silica and aluminum oxide nanoparticles showed higher heat curing ratios than inorganic silica particles due to the high compatibility with organic resins and excellent particle dispersion in polymer matrix. Notably, aluminum oxide-based hybrid nanoparticles exhibited higher heat curing ratio than the silica-based nanoparticles, which is in accordance with previous result that the aluminum oxide filler acted as a catalyst for epoxy–amine coupling reaction and thus increased the initial reaction velocity of the epoxy curing reactions [21].

### 3.4. Adhesion Properties of UV/Heat Dual-Curable Epoxy–Acrylate Composites

**Figure 9:**
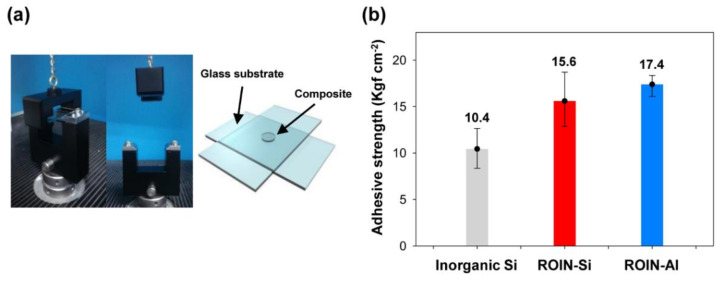
(**a**) Measurement of adhesion strength using pull-off jig and glass substrates. (**b**) Adhesive strengths of the epoxy–acrylate composites containing inorganic silica or reactive hybrid nanoparticles.

The adhesive strength of the epoxy–acrylate composites was analyzed using glass substrates and pull-off jig, as shown in Figure 9a. The sealing composite was applied between two glass substrates, and then cured by UV and heat treatment. Figure 9b shows the adhesion strength of epoxy–acrylate composites containing inorganic silica or reactive hybrid nanoparticles. Since the inorganic silica particles are not sufficiently compatible with organic resins in the composite, a relatively low adhesion strength of 10.4 Kgf cm^−2^ was obtained for conventional composite. In contrast, the organic–inorganic hybrid silica or aluminum oxide nanoparticles with an organic surface of methacrylate groups showed improved adhesive strengths of 15.6 and 17.4 Kgf cm^−2^, respectively. Moreover, cohesive failure was found for all epoxy–acrylate composites. It is supposed that the enhanced resin compatibility and high curing ratio of the proposed composites induce densely photocrosslinked network between reactive hybrid nanoparticles and matrix resins, leading to the improvement in the adhesion strength. Moreover, the methacrylate groups in the surface of nanoparticles offer high adhesive strength and low shrinkage compared to the acrylate groups [35,36]. Notably, the epoxy–acrylate composite including reactive aluminum oxide nanoparticles exhibited a relatively high adhesion strength due to the high UV and heat curing ratios compared to that of the composite with reactive silica nanoparticles.

### 3.5. Moisture Permeability Performance of UV/Heat Dual-Curable Epoxy–Acrylate Composites

**Figure 10:**
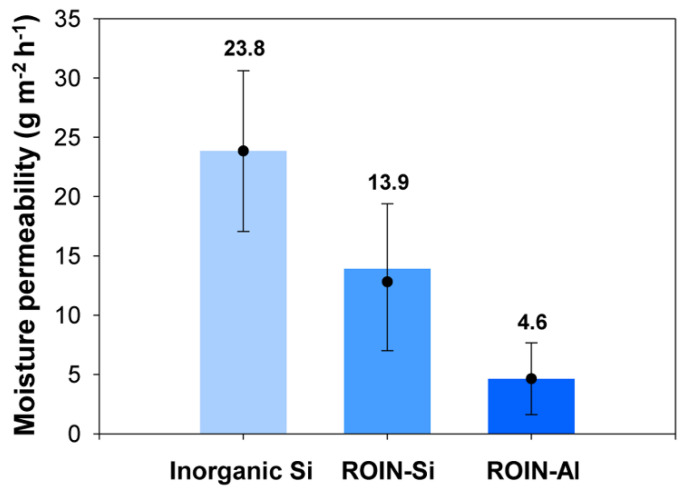
Moisture permeability of the epoxy–acrylate composites containing inorganic silica or reactive hybrid nanoparticles.

Figure 10 shows the moisture permeability of the prepared epoxy–acrylate composites including inorganic silica or reactive hybrid nanoparticles. The moisture permeability is defined as Equation (2).

(2) $$\mathrm{Moisture}\;\mathrm{permeability}\;\left(\%\right)=\frac{final\;weight\;of\;drierite\;-\;initial\;weight\;of\;drierite}{\left(drawing\;area\;of\;composite\right)\times \left(storage\;time\;in\;water\right)}\times 100$$

The epoxy–acrylate composite containing inorganic silica particles showed a relatively high moisture permeability of 23.8 g m^−2^ h^−1^, which appears to be the result of low resin compatibility and low curing ratios. The decrease in water barrier performance of the sealing composite can lead to the remarkable deterioration in the operation reliability of the display device. On the contrary, the improved water-resistant performance was achieved for the proposed composites with reactive hybrid nanoparticles. The silica and aluminum oxide nanoparticles endowed the composites with relatively low moisture permeability of 13.9 and 4.6 g m^−2^ h^−1^, respectively. The creation of a firm crosslinked nanoparticle network with matrix resins afforded water-resistant property for the proposed composites due to high curing ratios of methacrylate and epoxy groups. Compared to the water permeability of reactive silica nanoparticles, the photoreactive aluminum oxide nanoparticles showed a more improved water barrier property owing to the formation of densely crosslinked network.

## 4. Conclusions

In this study, a new type of UV/heat dual-curable epoxy–acrylate composite with high adhesion strength and excellent water barrier performance, a promising sealing material in narrow bezel devices, has been prepared using reactive organic–inorganic hybrid silica and aluminum oxide nanoparticles. The reactive hybrid nanoparticles comprising the core of silica or aluminum oxide and the shell of methacrylate groups were synthesized simply through sol–gel coupling reaction and a chemisorption process. The chemical structure, morphology, and particle size of the synthesized hybrid nanoparticles were confirmed by FTIR spectroscopy, FE-SEM, and Zetasizer. The newly prepared epoxy–acrylate composites consisting of reactive resins, photoinitiator, hardener, and reactive nanoparticles exhibited a relatively low viscosity of about 20,000 cP due to the high compatibility with organic resin and nanoscale particle size. Moreover, narrow and uniform dispensing lines with an average line width of about 1.4 mm were observed for the proposed composites. The curing behavior of the newly prepared composites was different from that of the conventional composite containing inorganic silica particles. The proposed composites with hybrid silica or aluminum oxide nanoparticles showed extremely high UV curing ratios of 94.5% and 98.1%, respectively, owing to the presence of a large number of methacrylate functional groups in the surface of nanoparticles. In the case of heat curing ratio, aluminum oxide-based hybrid nanoparticles exhibited a relatively high heat curing ratio of 87% due to the catalytic effect of aluminum oxide on the epoxy–amine coupling reaction. The sealing composites with surface-modified nanoparticles exhibited the enhanced adhesive strength due to the formation of a photocrosslinked nanoparticle network with matrix resins. In particular, the composite containing reactive aluminum oxide nanoparticles exhibited a relatively high adhesive strength due to the high UV and heat curing ratios compared to that of the composite including silica nanoparticles. In addition, the proposed sealing composite with reactive aluminum oxide nanoparticles showed an excellent water-resistant property of 4.6 g m^−2^ h^−1^ owing to the creation of firm crosslinked nanoparticle network with matrix resins. Our epoxy–acrylate sealing composites containing photoreactive organic–inorganic hybrid nanoparticles are expected to be useful for applications in narrow bezel devices and functional adhesive materials for water-resistant coatings and pressure-sensitive adhesives owing to the exceptional properties of low viscosity, narrow dispensing width, high curing ratios, excellent adhesion strength, and low moisture permeability.

## Figures and Tables

**Table 1:** Composition of UV/heat dual-curable epoxy–acrylate composites.

Composition	Chemical Compound	Conventional Composite (wt.%)	New Epoxy–Acrylate Composites with Reactive Nanoparticles (wt.%)	
Resin	YD-128	40	40	40
	BisGMA	40	40	40
Hardener	ADH	1.5	1.5	1.5
Photoinitiator	Irgacure 651	2.5	2.5	2.5
Filler	Fumed silica	16	-	-
	Reactive hybrid silica nanoparticles (ROIN-Si)	-	16	-
	Reactive hybrid aluminum oxide nanoparticles (ROIN-Al)	-	-	16
